# Dental Anxiety in Pediatric Patients: Contemporary Assessment and Multimodal Management Strategies

**DOI:** 10.3390/children13030397

**Published:** 2026-03-12

**Authors:** Roxana Alexandra Cristea, Ioana Scrobota, Mihail Pantor, Liliana Sachelarie, Gabriela Ciavoi

**Affiliations:** 1 Doctoral School of Biomedical Science, University of Oradea, 410087 Oradea, Romania; 2 Department of Dental Medicine, Faculty of Medicine and Pharmacy, University of Oradea, 410073 Oradea, Romania; ioana_scrobota@uoradea.ro (I.S.); mpator@uoradea.ro (M.P.); gciavoi@uoradea.ro (G.C.); 3 Preclinical Sciences Department, Faculty of Medicine, Apollonia University, 700511 Iasi, Romania

**Keywords:** dental anxiety, pediatric dentistry, behavioral management, anxiety assessment tools, virtual reality, audiovisual distraction, child behavior

## Abstract

**Highlights:**

**What are the main findings?**

- Validated pediatric dental anxiety assessment tools differ in structure and applicability, requiring age-appropriate selection in clinical practice.
- Contemporary non-pharmacological strategies, including communication techniques, environmental modifications, and digital distraction, effectively improve cooperation and reduce anxiety levels.

**What are the implications of the main findings?**

- Structured anxiety assessment combined with multimodal behavioral strategies enhances treatment outcomes and clinical efficiency in pediatric dentistry.
- The integration of emerging digital technologies, such as immersive distraction methods, represents a promising direction for modern anxiety management.

**Abstract:**

**Background**: Dental anxiety remains a prevalent and persistent challenge in pediatric dentistry, significantly affecting children’s cooperation, treatment outcomes, and long-term oral health behaviors. Despite advances in minimally invasive care, anxiety continues to act as a barrier to effective clinical management. This narrative review aims to synthesize current evidence on validated assessment tools for pediatric dental anxiety and to examine contemporary non-pharmacological management strategies applicable in routine clinical practice. **Methods**: A structured literature search was conducted in major electronic databases to identify relevant studies, systematic reviews, and clinical guidelines addressing dental anxiety assessment and behavioral management in children. Particular emphasis was placed on validated anxiety scales, communication strategies, environmental adaptations, and emerging digital interventions such as immersive distraction technologies. **Results**: Multiple validated instruments are available to assess pediatric dental anxiety; however, their applicability varies by age, cognitive development, and clinical context. Non-pharmacological approaches including tell–show–do, modeling, parental guidance, audiovisual distraction, and virtual reality-based techniques demonstrate consistent effectiveness in reducing anxiety and improving behavioral cooperation. Recent trends emphasize multimodal, patient-centered strategies integrating communication, environmental modification, and digital tools. **Conclusions**: Structured anxiety assessment combined with contemporary multimodal management strategies can enhance clinical efficiency, improve child cooperation, and promote positive dental experiences. The integration of emerging digital technologies represents a promising advancement in pediatric anxiety management and supports a more individualized approach to care. Furthermore, a structured multimodal clinical framework is proposed to facilitate chairside decision-making and practical implementation.

## 1. Introduction

Dental anxiety represents a significant and persistent challenge in pediatric dentistry, affecting a substantial proportion of children worldwide. Epidemiological studies report that the prevalence of dental anxiety in children ranges between 10% and 20%, with higher values observed in younger age groups and in children with previous negative dental experiences [1,2]. Despite advances in preventive, and minimally invasive dentistry, dental anxiety remains a major barrier to the effective delivery of oral healthcare in pediatric populations.

Dental anxiety in children is a multifactorial phenomenon, influenced by individual psychological traits, developmental stage, parental anxiety, and environmental factors within the dental office [3,4]. Children often lack the cognitive and emotional maturity required to understand dental procedures, which may lead to exaggerated fear responses to unfamiliar instruments, sounds, or sensations. Moreover, parental attitudes and behaviors have been shown to play a crucial role, as anxious parents may inadvertently transmit their fears to their children [5].

The clinical impact of dental anxiety extends beyond emotional distress. Elevated anxiety levels are strongly associated with reduced cooperation, avoidance of dental visits, and incomplete or postponed treatments [6]. As a consequence, anxious pediatric patients are at increased risk of untreated caries, progression of oral disease, and the need for more complex or invasive interventions over time [7]. From a clinical perspective, poor cooperation related to anxiety can compromise treatment quality, prolong chair time, and increase stress for both the dental practitioner and the child.

Effective management of dental anxiety is therefore essential to ensure successful treatment outcomes and foster positive long-term attitudes toward oral healthcare. While pharmacological approaches such as sedation or general anesthesia may be indicated in selected cases, they are not without risks and are not suitable for routine use in the majority of pediatric patients [8]. Furthermore, reliance on pharmacological methods does not address the underlying psychological mechanisms of anxiety and may fail to promote adaptive coping strategies.

In this context, non-pharmacological management strategies represent the cornerstone of anxiety control in pediatric dental practice. Behavioral techniques, communication strategies, and environmental modifications aim to reduce fear, enhance predictability, and improve the child’s sense of control during dental treatment [9,10]. These approaches are non-invasive, cost-effective, and can be readily integrated into routine clinical workflows. Importantly, they contribute not only to immediate cooperation but also to the development of positive dental experiences that may persist into adulthood.

Given the high prevalence of dental anxiety in children and its significant clinical implications, a structured approach to anxiety assessment and management is essential. Understanding how to identify anxious pediatric patients and apply appropriate non-pharmacological strategies is a key competence for dental practitioners.

This narrative review aims to synthesize current evidence on dental anxiety in pediatric patients, with a particular focus on clinically applicable assessment tools and practical management strategies that can be implemented in everyday dental practice. In addition, this review proposes a structured multimodal clinical algorithm integrating validated assessment tools with contemporary behavioral and technology-assisted strategies to support practical application in pediatric dental care.

## 2. Materials and Methods

This article presents a narrative review based on a focused literature search conducted in the PubMed, Scopus, and Web of Science databases. The search aimed to identify clinically relevant studies, systematic reviews, and clinical guidelines addressing pediatric dental anxiety assessment and non-pharmacological management strategies. To emphasise contemporary clinical approaches, priority was given to studies published between 2007 and 2025. Earlier seminal publications were selectively included when relevant to foundational theoretical concepts or to the validation of widely used assessment instruments. This review does not follow a systematic review protocol but provides an integrative synthesis of current evidence to support clinical practice. Studies were further selected based on clinical relevance, methodological robustness, and their contribution to the contemporary understanding of pediatric dental anxiety assessment and behavioral management strategies. The selection process aimed to ensure conceptual coherence and practical applicability within routine pediatric dental practice.

## 3. Dental Anxiety in Pediatric Patients

Dental anxiety in pediatric patients is a complex psychological response characterized by fear, apprehension, or distress associated with dental environments and procedures. In children, dental anxiety differs from that observed in adults due to developmental, cognitive, and emotional factors that influence perception and coping mechanisms. Younger children, in particular, may have difficulty distinguishing between real and perceived threats, which can intensify anxiety reactions during dental visits [11].

The prevalence of dental anxiety among children and adolescents has been widely reported in the literature, with estimates ranging from 10% to over 20%, depending on age, assessment method, and cultural context [11,12]. Higher levels of anxiety are frequently observed in preschool and early school-aged children, as well as in those with previous negative dental or medical experiences. Importantly, dental anxiety in childhood may persist over time and extend into adolescence and adulthood if not appropriately managed [12].

Dental anxiety in pediatric patients is multifactorial in origin. Individual characteristics, including temperament, emotional sensitivity, and general anxiety traits, contribute significantly to fear responses. Additionally, parental anxiety and family attitudes toward dental care play a critical role, as children may model fearful behaviors observed in their caregivers [13]. Negative verbal communication or expressions of concern by parents prior to dental visits have been shown to increase anxiety levels in children.

The dental environment itself represents another important contributor to pediatric dental anxiety. Unfamiliar instruments, high-pitched sounds, fear of pain, and a perceived lack of control are commonly reported triggers [14]. Ineffective communication between the dental practitioner and the child, particularly when explanations are not adapted to the child’s developmental level, may further exacerbate anxiety and lead to behavioral management problems, such as refusal of treatment or avoidance of dental care.

A clear understanding of the prevalence, determinants, and clinical manifestations of dental anxiety in children is essential for effective management. Early recognition of anxious pediatric patients allows dental practitioners to adapt communication strategies and implement appropriate non-pharmacological interventions, thereby improving cooperation and promoting positive long-term attitudes toward oral healthcare.

### 3.1. Definition and Conceptual Framework

Dental anxiety in children is defined as an excessive emotional response characterized by apprehension, tension, or fear related to dental situations, whether real or anticipated. It represents a multifaceted psychological state that may occur before, during, or even in anticipation of dental treatment. In pediatric patients, dental anxiety is particularly influenced by developmental factors, including limited cognitive capacity to understand procedures and reduced ability to regulate emotional responses [15].

It is important to distinguish between dental fear and dental anxiety, as these concepts, although closely related, are not interchangeable. Dental fear is generally considered an immediate emotional reaction to a specific, identifiable stimulus, such as a dental instrument, sound, or perceived pain. In contrast, dental anxiety refers to a more diffuse and persistent state of apprehension, often arising in anticipation of dental treatment, even in the absence of direct stimuli [16]. Anxiety is therefore more strongly associated with avoidance behaviors and long-term negative attitudes toward dental care.

In pediatric populations, dental anxiety often develops as a result of both direct and indirect learning experiences. Direct experiences may include previous painful or unpleasant dental procedures, while indirect experiences encompass observational learning, parental modeling, and negative information received from family members or peers [17]. Children are particularly susceptible to these influences because they rely on caregivers for emotional cues and for interpreting unfamiliar situations.

Pediatric dental anxiety also presents distinct characteristics compared to anxiety in adults. Children frequently express anxiety through behavioral manifestations rather than verbal communication, including crying, refusal of treatment, withdrawal, or aggressive behavior. These responses reflect immature coping mechanisms and limited emotional self-regulation [18]. Additionally, the child’s developmental stage plays a critical role, as younger children tend to exhibit higher anxiety levels due to reduced predictability, lack of perceived control, and heightened sensitivity to sensory stimuli within the dental environment.

Understanding dental anxiety in children within a conceptual framework that integrates emotional, cognitive, behavioral, and social components is essential for effective clinical management. Recognizing the distinction between fear and anxiety, as well as the developmental and contextual factors unique to pediatric patients, provides the foundation for appropriate assessment and targeted non-pharmacological management strategies in pediatric dental practice.

### 3.2. Prevalence and Risk Factors

The prevalence of dental anxiety in pediatric populations varies considerably across studies, influenced by age, cultural background, assessment instruments, and study design. Epidemiological data consistently indicate that dental anxiety affects a substantial proportion of children, with reported prevalence rates ranging from approximately 10% to over 25% in different pediatric cohorts [18,19]. Higher anxiety levels are generally observed in younger children, particularly during early childhood, when cognitive immaturity and limited understanding of dental procedures contribute to heightened fear responses.

Age represents one of the most significant determinants of dental anxiety in children. Younger patients tend to exhibit increased anxiety due to reduced coping abilities, limited perceived control, and heightened sensitivity to sensory stimuli such as sounds and vibrations [18]. As children grow older and accumulate dental experiences, anxiety levels may decrease; however, this trend is not universal and largely depends on the nature of prior dental encounters [19].

Previous negative dental or medical experiences constitute a major risk factor for the development of dental anxiety. Painful or traumatic treatments, especially those occurring at an early age, have been strongly associated with persistent fear and avoidance behaviors [20]. Conversely, positive early dental visits characterized by effective communication and minimal discomfort may reduce the likelihood of anxiety development.

Parental anxiety has been identified as another critical contributor to pediatric dental anxiety. Children often model emotional responses on those of their caregivers, and anxious parents may unintentionally convey fear through verbal expressions, body language, or anticipatory warnings before dental appointments [21]. This intergenerational transmission of anxiety highlights the importance of involving parents in anxiety management strategies.

Additional risk factors include temperament traits, such as behavioral inhibition and general anxiety proneness, as well as the environmental characteristics of the dental clinic, including unfamiliar equipment, clinical smells, and lack of child-friendly design [22,23]. Socio-cultural factors and previous exposure to negative information about dental care may further amplify anxiety levels in pediatric patients, Table 1.

Overall, dental anxiety in children arises from the interaction of individual, familial, and environmental factors. Identifying these risk factors is essential for early detection of vulnerable patients and for tailoring preventive and management strategies within pediatric dental practice.

## 4. Assessment of Dental Anxiety in Children

The assessment of dental anxiety in pediatric patients represents a fundamental step in planning effective behavior management strategies. Early identification of anxious children allows dental practitioners to adapt communication, select appropriate non-pharmacological techniques, and prevent the escalation of fear-related behaviors during dental treatment. Given the subjective nature of anxiety, the use of structured and validated assessment tools is essential for obtaining reliable and reproducible information in clinical practice [24].

Unlike adult patients, children often cannot articulate their emotional states verbally. As a result, dental anxiety in pediatric populations is frequently expressed through behavioral manifestations rather than self-reported feelings. For this reason, assessment tools used in pediatric dentistry typically rely on behavioral observation scales, visual self-report instruments, or caregiver-assisted questionnaires, each adapted to the child’s developmental level [25].

Several assessment instruments have been validated for use in pediatric dental settings. Among the most commonly used are the Frankl Behavior Rating Scale, the Facial Image Scale (FIS), and the Children’s Fear Survey Schedule–Dental Subscale (CFSS-DS). These tools differ in complexity, administration methods, and clinical applicability, but all provide valuable information about the child’s anxiety level and cooperation during dental visits [26,27,28].

Behavioral observation scales, such as the Frankl scale, focus on the child’s observable reactions during dental treatment, offering immediate and practical insights for chairside decision-making. In contrast, self-report measures like the FIS allow children to express their emotional state using visual cues, making them particularly suitable for younger age groups, Table 2. Questionnaire-based instruments, such as the CFSS-DS, provide a more comprehensive evaluation of dental fear by assessing multiple fear-related stimuli, although their use may be limited by time constraints in routine practice [27,28].

When selecting an appropriate anxiety assessment tool, consider the child’s age, cognitive development, and communication abilities and the clinical context. In many cases, combining behavioral observation with a simple self-report measure enhances assessment accuracy and supports individualized anxiety management planning.

## 5. Management of Dental Anxiety in the Dental Office

Effective management of dental anxiety in pediatric patients is essential to ensure successful treatment outcomes and positive long-term attitudes toward oral health care. Contemporary pediatric dentistry emphasizes non-pharmacological behavior guidance techniques as first-line strategies, given their safety, adaptability, and potential to address the underlying psychological mechanisms of anxiety [29]. These approaches aim to reduce fear, enhance cooperation, and promote a sense of control in the child during dental treatment.

### 5.1. Behavioral Management Techniques

Among the most widely used behavioral techniques is the tell–show–do method, which involves verbal explanation, demonstration of procedures, and subsequent performance in a non-threatening manner. This technique has been shown to reduce uncertainty and fear by increasing predictability and familiarity with dental procedures [30]. Positive reinforcement, including verbal praise and rewards, further strengthens cooperative behavior and encourages adaptive coping responses.

Modeling represents another effective strategy, particularly in younger children. Observational learning, in which a child watches a cooperative peer or sibling undergo dental treatment, has been associated with reduced anxiety and improved compliance [31]. Distraction techniques, such as audiovisual aids, storytelling, or guided imagery, may also help divert attention away from anxiety-provoking stimuli during dental procedures.

**Figure 1:**
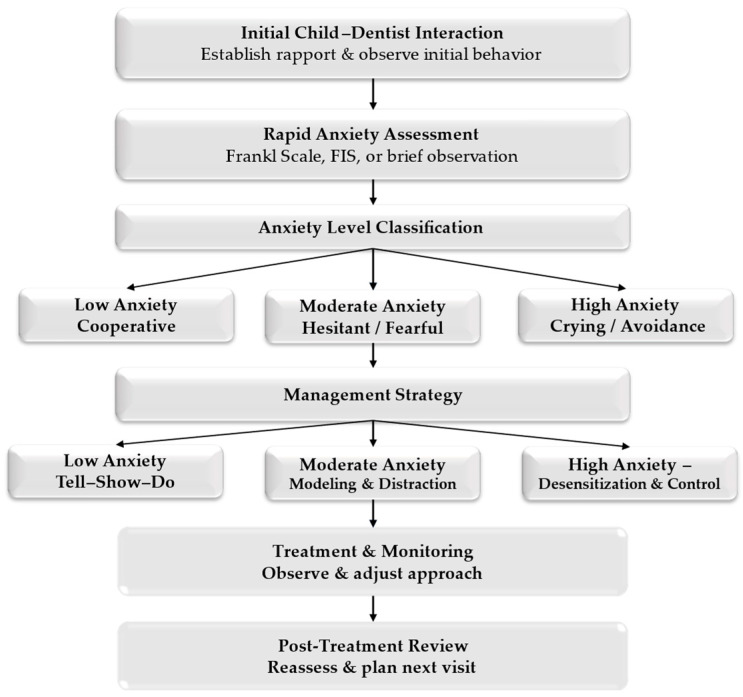
Chairside Management Algorithm for Dental Anxiety in Pediatric Patients.

To facilitate the practical application of these behavioral techniques in routine pediatric dental care, a structured chairside management algorithm is proposed (Figure 1).

In specific clinical circumstances where immediate treatment is required or when the child presents with severe behavioral dysregulation that compromises safety, advanced behavior guidance techniques such as passive or active protective stabilization may be considered. According to current guidelines of the American Academy of Pediatric Dentistry, protective stabilization should be applied only when clearly justified, with informed parental consent, appropriate documentation, and careful ethical consideration. These techniques are not first-line strategies but may function as adjunctive measures within a comprehensive and individualized behavior management framework.

### 5.2. Communication Strategies

Effective communication is a central pillar in managing dental anxiety in pediatric patients, as it directly influences the child’s perception of safety, predictability, and control during dental treatment [32]. Unlike adults, children rely heavily on verbal tone, non-verbal cues, and contextual framing to interpret potentially threatening situations. Therefore, communication in pediatric dentistry must be developmentally adapted, emotionally supportive, and strategically structured.

Age-appropriate language is essential. Technical terminology should be replaced with neutral or positive descriptors (e.g., “cleaning sugar bugs” instead of “removing decay”), while avoiding words associated with pain or threat. The use of simple, concrete explanations enhances cognitive understanding and reduces anticipatory anxiety. Additionally, short, sequential information delivery prevents cognitive overload and helps maintain the child’s attention.

Voice control and nonverbal communication also play significant roles. A calm tone, a steady rhythm, eye-level positioning, and reassuring facial expressions reinforce a sense of trust and authority. Studies indicate that congruence between verbal explanations and non-verbal cues enhances emotional regulation in anxious pediatric patients.

Providing limited, structured choices is another effective strategy to enhance the child’s perceived sense of control. Allowing the child to select between two acceptable options such as choosing the color of a bib, selecting background music, or deciding which tooth to treat first supports autonomy without compromising clinical workflow. Research suggests that even minor decision-making opportunities can significantly reduce situational anxiety.

Communication strategies should also incorporate anticipatory guidance. Preparing the child for upcoming sensations (e.g., “You may feel some vibration”) reduces uncertainty and improves coping responses. Predictability is a key protective factor against anxiety escalation.

Parental involvement represents a complex but important dimension of communication. While parental presence may provide reassurance for some children, it may increase anxiety in others, particularly when parents exhibit visible fear, overprotectiveness, or intrusive behaviors. Dental practitioners should assess the emotional dynamic between parent and child and adapt communication accordingly. In some cases, brief parental counseling prior to treatment can improve the overall emotional climate in the operatory [33].

Collectively, structured communication techniques not only reduce immediate anxiety but also contribute to the formation of positive cognitive schemas associated with dental care, supporting long-term behavioral adaptation. The principal communication strategies and their psychological mechanisms are summarized in Table 3.

### 5.3. Environmental and Sensory Modifications

The physical and sensory characteristics of the dental environment play a critical role in shaping the emotional and behavioral responses of pediatric patients. For many children, the dental clinic represents an unfamiliar and potentially threatening setting, characterized by intense sensory stimuli such as bright lights, high-frequency sounds, clinical smells, and the sight of metallic instruments. These environmental factors may activate anticipatory fear responses even before treatment begins [34].

Child-centered clinic design has therefore become an important component of anxiety management in pediatric dentistry. The use of calming color palettes, playful wall decorations, natural lighting, and visually familiar imagery can reduce environmental threat perception and promote emotional comfort. Waiting areas designed with child-appropriate furniture, toys, or interactive elements may facilitate gradual adaptation to the clinical setting before treatment initiation.

Auditory stimuli represent another major anxiety trigger. High-pitched sounds from dental handpieces are frequently associated with fear in children. Noise reduction strategies, including sound-dampening materials, quieter equipment, or the use of background music, have been shown to decrease physiological markers of anxiety. Music therapy, in particular, may promote relaxation by modulating autonomic nervous system responses and reducing heart rate variability associated with stress [35,36,37,38].

Audiovisual distraction techniques are among the most widely used sensory adaptations in contemporary pediatric dental practice. The use of ceiling-mounted screens, cartoons, virtual reality devices, or interactive storytelling can redirect the child’s attention away from potentially anxiety-provoking stimuli. By engaging cognitive resources in alternative tasks, distraction techniques reduce pain perception and procedural awareness, thereby improving behavioral cooperation [39,40,41,42,43,44,45].

Virtual reality (VR)-based distraction systems represent one of the most promising technological advancements in pediatric dental anxiety management. By immersing the child in an interactive, three-dimensional virtual environment, VR reduces awareness of dental stimuli and significantly decreases subjective anxiety levels and physiological stress indicators compared with conventional techniques [46,47].

Virtual reality interventions may be broadly categorized into passive systems, in which the child observes a virtual environment without interactive control, and active immersive systems that allow user interaction within a three-dimensional setting. Active VR systems appear to reduce anxiety more effectively through enhanced cognitive engagement and attentional absorption, thereby limiting the processing of anxiety-provoking procedural stimuli [46,47]. Randomized controlled trials have demonstrated significant reductions in self-reported anxiety scores and physiological parameters such as heart rate in children exposed to immersive VR during restorative procedures compared with conventional behavioral techniques [46]. Furthermore, recent reviews emphasize that immersive digital distraction represents one of the most promising contemporary adjuncts in pediatric anxiety management when integrated within multimodal behavioral frameworks [47]. However, practical considerations, including equipment cost, infection control protocols, patient age, and tolerance to head-mounted devices, should be evaluated before routine clinical implementation.

Immersive distraction techniques extend beyond passive entertainment by actively engaging multiple sensory modalities, including visual, auditory, and, in some systems, interactive motor responses. This deep cognitive engagement reduces the salience of anxiety-provoking stimuli within the dental environment and promotes emotional regulation. Evidence suggests that immersive systems may be particularly beneficial for children with moderate-to-high baseline anxiety levels, as they facilitate temporary psychological detachment from procedural stressors [46,47].

In addition to visual and auditory adaptations, tactile and olfactory modifications may further contribute to anxiety reduction. Weighted blankets, soft textures, or aromatherapy with mild, non-clinical scents may enhance the child’s sense of safety and comfort. Although evidence regarding these interventions is still emerging, preliminary findings suggest that multi-sensory approaches may be particularly beneficial in children with heightened sensory sensitivity or neurodevelopmental conditions.

Importantly, environmental modifications should not be regarded as isolated interventions but rather as components of an integrated anxiety management framework. When combined with structured communication strategies and behavioral guidance techniques, sensory adaptations create a supportive clinical atmosphere that facilitates emotional regulation and positive behavioral learning.

Collectively, environmental and sensory modifications are effective, low-risk, and easy-to-implement strategies for managing dental anxiety in children. Their consistent application, alongside validated anxiety assessment tools, contributes to improved cooperation, shorter treatment times, enhanced parental satisfaction, and the development of positive long-term attitudes toward dental care. The principal environmental and sensory interventions are summarized in Table 4.

Beyond environmental and sensory modifications, recent years have witnessed the increasing integration of digital and technology-assisted interventions into pediatric dental anxiety management, reflecting a shift toward more interactive and personalized care models [46,47].

### 5.4. Digital and Technology-Integrated Approaches

The integration of digital technologies into pediatric dental practice represents an important evolution in anxiety management strategies. Beyond traditional behavioral guidance techniques, interactive digital tools are increasingly used to enhance engagement, perceived control, and procedural cooperation in children.

It is important to distinguish immersive virtual reality (VR) systems from conventional audiovisual distraction delivered through ceiling-mounted screens or tablets [46,47]. Traditional audiovisual distraction remains a predominantly passive modality, in which the child maintains visual and peripheral awareness of the dental environment [47]. In contrast, immersive VR head-mounted displays create a three-dimensional visual field that substantially reduces environmental visual input and enhances sensory isolation [46]. This immersive quality limits exposure to anxiety-provoking stimuli such as dental instruments, overhead lights, and clinical movement, thereby facilitating deeper attentional absorption and more pronounced cognitive disengagement from the procedural context [46,47]. Consequently, immersive VR may exert stronger anxiolytic effects compared with standard screen-based distraction in children with moderate-to-high baseline anxiety levels [46].

Virtual reality-based systems are among the most extensively studied digital interventions. Randomized controlled trials have demonstrated that immersive VR distraction significantly reduces anxiety and pain perception during dental procedures compared with conventional approaches [46]. A recent systematic review further confirmed the effectiveness of audiovisual and immersive distraction techniques in improving behavioral outcomes in pediatric patients [47].

In addition to VR, tablet-based applications and gamified behavioral platforms have been introduced as adjunctive tools to facilitate emotional preparation and procedural familiarization. These digital resources allow children to engage with age-appropriate interactive content before or during treatment, thereby reducing anticipatory anxiety and enhancing coping capacity [45]. Such tools may also improve communication between clinician and patient by translating clinical steps into structured, child-friendly narratives.

Importantly, digital integration does not replace established behavioral guidance techniques but rather complements them within a multimodal management framework. By combining structured anxiety assessment, developmentally adapted communication, environmental modifications, and immersive digital distraction, clinicians can deliver more individualized and psychologically informed care.

Emerging evidence suggests that immersive digital interventions may be particularly beneficial for children presenting with moderate-to-high baseline anxiety, as enhanced cognitive engagement facilitates temporary psychological detachment from procedural stressors [46,47]. However, further high-quality longitudinal studies are needed to evaluate long-term behavioral outcomes and cost-effectiveness.

Despite promising clinical outcomes, immersive VR systems present several practical limitations that must be considered before routine implementation [46,47]. Equipment costs may represent a barrier for smaller dental practices, particularly when high-quality head-mounted devices and compatible software platforms are required. Additionally, some children may experience discomfort, claustrophobic reactions, or reluctance to wear headsets, particularly at younger ages or in children with sensory sensitivity [46]. Infection control protocols require strict disinfection procedures between patients, and device hygiene remains an essential consideration in pediatric settings. Furthermore, prolonged headset use may not be suitable for lengthy procedures or for patients requiring continuous verbal interaction [47]. Therefore, VR should be viewed as an adjunctive tool within a broader multimodal framework rather than a universal replacement for established behavioral guidance techniques [46,47].

## 6. Clinical Implications and Practical Application

Contemporary anxiety management increasingly adopts multimodal frameworks that combine structured assessment, developmentally adapted communication, environmental modification, and technology-assisted distraction within a unified clinical pathway. Rather than relying on isolated behavioral techniques, this integrative model allows clinicians to tailor interventions according to the child’s anxiety level, cognitive maturity, and emotional profile. Multimodal strategies enhance clinical flexibility and reflect a patient-centered approach to pediatric dental care.

The effective management of dental anxiety in pediatric patients relies on the translation of evidence-based behavior guidance principles into practical, chairside decision-making. The structured management model proposed in this review supports dental practitioners in identifying anxiety levels early and selecting appropriate non-pharmacological strategies tailored to each child’s developmental and emotional profile [38].

In routine clinical practice, the incorporation of rapid anxiety assessment tools enables early recognition of anxious behaviors before they escalate into treatment resistance or avoidance. Simple observational scales and visual self-report instruments can be implemented quickly and provide valuable guidance for adjusting communication style, treatment pacing, and environmental adaptations [39]. This proactive approach improves workflow efficiency and reduces stress for both the child and the dental team.

The proposed algorithm emphasises individualised and flexible management rather than rigid protocol-based care. Children with low anxiety levels may be managed effectively using standard behavioral techniques, while those with moderate anxiety benefit from enhanced communication strategies, modeling, and distraction techniques. For children exhibiting high anxiety levels, the model encourages gradual desensitization, short treatment sessions, and careful scheduling of invasive procedures [40].

An important clinical implication of this framework is the promotion of continuity of care. Reassessment of anxiety levels and reinforcement of positive behavior at the end of each visit allow dental practitioners to monitor progress over time and adapt management strategies accordingly. Evidence suggests that repeated positive dental experiences during childhood can significantly reduce anxiety and improve cooperation during future dental visits [41], as shown in Table 5.

The practical application of non-pharmacological anxiety management strategies has been associated with improved treatment outcomes, including enhanced patient cooperation, reduced appointment duration, and increased parental satisfaction. Furthermore, these approaches align with contemporary recommendations advocating child-centered, minimally invasive, and psychologically informed dental care [42].

Overall, the proposed clinical model serves as a practical decision-support framework that bridges the gap between theoretical concepts and everyday pediatric dental practice. Its adaptability makes it suitable for implementation in both general dental offices and specialized pediatric clinics, contributing to safer, more effective, and more positive dental care experiences for children.

## 7. Discussion

Dental anxiety in pediatric patients remains a prevalent and clinically significant challenge, with direct implications for treatment delivery, patient cooperation, and long-term oral health behaviors. The literature consistently indicates that dental anxiety during childhood is multifactorial, involving developmental vulnerability, previous negative dental experiences, parental influence, and environmental factors within the dental setting [42,43].

The variability in reported prevalence rates of pediatric dental anxiety across studies reflects important methodological heterogeneity. Differences in assessment tools, age ranges, cultural contexts, and operational definitions of fear versus anxiety complicate direct comparisons between cohorts. While some instruments capture situational fear responses, others evaluate broader trait-related anxiety patterns, potentially measuring distinct psychological constructs. Moreover, the absence of universal cut-off thresholds and inconsistent reporting standards further contribute to variability in epidemiological data. This heterogeneity highlights the need for greater standardization in both measurement instruments and reporting frameworks to improve comparability and strengthen evidence-based recommendations.

A central aspect of this review is the importance of early, structured assessment of dental anxiety. Several studies emphasize that failure to recognize anxiety at an early stage may lead to escalating behavioral management problems, appointment cancellations, and avoidance of dental care [44]. The routine use of validated assessment tools enables clinicians to identify anxiety-related behaviors before invasive procedures are initiated, thereby facilitating preventive and individualized management.

The management strategies discussed in this review reinforce the role of non-pharmacological behavior guidance techniques as the cornerstone of pediatric dental anxiety control. Techniques such as tell–show–do, positive reinforcement, modeling, and distraction have demonstrated effectiveness in reducing fear and improving cooperation, while simultaneously supporting positive emotional learning [45]. These approaches align with current professional recommendations advocating child-centered and minimally invasive care.

An important contribution of this review is the integration of anxiety assessment and behavior management into a structured chairside clinical algorithm. Rather than treating individual techniques as isolated interventions, the proposed model offers a flexible decision-support framework that guides clinicians from identifying anxiety to selecting strategies and conducting continuous reassessment. This integrative approach reflects the dynamic nature of dental anxiety and accommodates individual variability among pediatric patients [43,44].

The clinical relevance of this framework lies in its adaptability to routine dental practice. By stratifying anxiety levels and linking them to tailored non-pharmacological interventions, the model supports efficient clinical decision-making while minimizing reliance on pharmacological methods. Moreover, the emphasis on reassessment and reinforcement across successive visits acknowledges the longitudinal nature of pediatric dental care and the potential for gradual reduction in anxiety over time [45].

Several limitations should be acknowledged. As a narrative review, this work does not provide quantitative synthesis or causal inference. Additionally, the proposed clinical model has not been prospectively validated and should be regarded as a conceptual and practical framework rather than a standardized protocol. Future studies should investigate the clinical effectiveness of integrated assessment–management models and explore their impact on long-term behavioral and oral health outcomes [45].

Although non-pharmacological strategies represent the cornerstone of pediatric anxiety management, their effectiveness may vary depending on individual child characteristics. Children with neurodevelopmental disorders, heightened sensory sensitivity, or severe dental phobia may respond less predictably to conventional communication or distraction techniques. In such cases, behavioral interventions alone may be insufficient, and adjunctive approaches including pharmacological support may be necessary. Additionally, audiovisual distraction techniques, while effective in reducing situational anxiety, do not necessarily address underlying cognitive fear schemas. These considerations underscore the importance of individualized assessment and flexible management planning rather than reliance on a single behavioral modality.

Overall, the findings discussed in this review highlight the critical role of structured assessment and individualized non-pharmacological management in pediatric dentistry. By bridging theoretical understanding with practical application, this approach contributes to improved cooperation, enhanced patient experience, and the development of positive attitudes toward dentistry in childhood.

In recent years, pediatric dental anxiety management has evolved from predominantly traditional behavioral guidance techniques toward more technologically integrated and multimodal approaches. While foundational methods such as tell–show–do, modeling, and parental guidance remain central to practice, contemporary advances increasingly incorporate digital tools and immersive technologies. Virtual reality (VR) distraction systems and interactive audiovisual platforms have shown promise in reducing procedural anxiety and perceived pain in pediatric patients [46,47]. Unlike passive distraction methods, immersive VR environments provide multisensory engagement, which may more effectively attenuate autonomic stress responses. Furthermore, modern anxiety management strategies are progressively conceptualized within multimodal frameworks that combine communication techniques, environmental modifications, behavioral guidance, and digital distraction into individualized care plans [45]. This integrative model reflects a shift from isolated interventions toward structured, patient-centered approaches tailored to developmental stage and psychological profile.

From a neurobiological perspective, emerging anxiety management strategies may exert their effects through the modulation of autonomic nervous system activity and attentional gating mechanisms. Immersive and distraction-based interventions, including virtual reality systems, have been shown to be associated with reductions in observable stress indicators such as heart rate and self-reported anxiety levels in pediatric dental settings [46,47]. Simultaneously, increased cognitive engagement limits the processing of threat-related stimuli through attentional modulation pathways, thereby reducing the subjective perception of fear and discomfort [15]. These mechanisms provide a theoretical and clinical rationale supporting the integration of immersive technologies into pediatric dental anxiety management.

While existing AAPD behavioral guidance guidelines provide comprehensive descriptions of individual management techniques [6], the present framework extends beyond a descriptive approach by integrating structured anxiety assessment with a stepwise, multimodal decision-making pathway. Unlike traditional models that primarily categorize techniques, this approach emphasizes the dynamic alignment between measured anxiety levels and tailored behavioral interventions, including contemporary digital strategies. By combining validated assessment tools with adaptable management protocols, the proposed framework aims to enhance clinical precision and facilitate individualized anxiety management in everyday pediatric dental practice.

This review has several limitations that should be acknowledged. As a narrative synthesis, it does not follow a systematic review protocol and may therefore be subject to selection bias and heterogeneity in study designs and outcome measures. The included evidence varies in methodological rigor, and direct quantitative comparisons between different anxiety management strategies were not performed. Additionally, emerging digital interventions such as immersive virtual reality remain supported by a limited number of high-quality randomized controlled trials, warranting cautious interpretation of their widespread clinical applicability.

Future research should focus on well-designed randomized controlled trials with standardized outcome measures to allow more robust comparisons between traditional and technology-assisted behavioral strategies. Longitudinal research has already demonstrated that early positive dental experiences and effective anxiety management are associated with improved long-term behavioral outcomes. Nevertheless, further studies are warranted to clarify the sustained impact of contemporary multimodal and technology-assisted interventions into adolescence and adulthood.

## 8. Conclusions

Dental anxiety in pediatric patients is a frequent and clinically significant issue that affects cooperation, treatment delivery, and long-term attitudes toward oral healthcare. Early identification of anxiety using age-appropriate assessment tools enables timely and individualized management.

Non-pharmacological behavior guidance techniques remain the foundation of pediatric dental anxiety management. When integrated into a structured chairside approach, these strategies support effective clinical decision-making and promote positive dental experiences. The proposed multimodal framework provides a practical, patient-centered decision-support model that integrates assessment, communication, environmental adaptation, and digital technologies within a unified clinical pathway.

Further studies are needed to evaluate the long-term effectiveness of integrated assessment and management models.

## Figures and Tables

**Table 1:** Major Risk Factors Associated with Dental Anxiety in Pediatric Patients.

Risk Factor	Description	Clinical Implications
Younger age	Limited cognitive understanding and emotional regulation	Increased need for behavioral guidance and predictability
Previous negative dental experiences	Painful or traumatic treatments during early visits	Higher risk of persistent anxiety and avoidance behaviors
Parental dental anxiety	Modeling of fear and negative expectations	Importance of parental counseling and reassurance
Temperament and general anxiety traits	Behavioral inhibition, emotional sensitivity	Need for individualized communication strategies
Dental environment	Unfamiliar sounds, instruments, smells	Benefit of child-friendly and sensory-adapted settings

**Table 2:** Commonly Used Tools for the Assessment of Dental Anxiety in Pediatric Patients.

Assessment Tool	Type of Assessment	Age Group	Main Advantages	Limitations
Frankl Behavior Rating Scale	Behavioral observation	All pediatric ages	Simple, rapid, chairside applicable	Subjective, observer-dependent
Facial Image Scale (FIS)	Self-report (visual)	3–10 years	Easy to understand, child-friendly	Limited emotional range
CFSS-DS	Questionnaire-based	≥6 years	Comprehensive assessment of fear stimuli	Time-consuming, requires cooperation

**Table 3:** Structured Communication Strategies in Pediatric Dental Anxiety Management.

Communication Strategy	Mechanism of Action	Clinical Example	Expected Psychological Effect
Age-appropriate language	Reduces cognitive uncertainty	“We are going to tickle the tooth”	Lower anticipatory fear
Voice control & calm tone	Emotional regulation through reassurance	Slow, steady speech	Increased trust
Limited structured choices	Enhances perceived control	Choosing music or treatment order	Reduced helplessness
Anticipatory guidance	Increases predictability	Explaining upcoming sensations	Improved coping
Positive framing	Replaces threat perception	Avoiding words like “pain” or “drill”	Lower threat appraisal
Tailored parental involvement	Modulates emotional transmission	Coaching parents before treatment	Stabilized emotional climate

**Table 4:** Environmental and Sensory Interventions in Pediatric Dental Anxiety Management.

Environmental Modification	Targeted Sensory Domain	Mechanism	Expected Clinical Benefit
Calming colors & decor	Visual	Reduces environmental threat perception	Improved emotional comfort
Background music	Auditory	Autonomic relaxation response	Lower physiological anxiety
Audiovisual distraction	Visual/Auditory	Cognitive redirection	Increased cooperation
Noise-reducing equipment	Auditory	Decreased sensory overload	Reduced anticipatory fear
Soft textures/weighted blankets	Tactile	Enhanced sense of security	Improved behavioral stability

**Table 5:** Clinical Mapping between Anxiety Level and Management Strategy in Pediatric Dental Patients.

Anxiety Level	Assessment Indicators	Recommended Management Approach	Expected Clinical Outcome
Low anxiety	Positive or mildly negative behavior; calm interaction	Tell–Show–Do, positive reinforcement	Smooth treatment, high cooperation
Moderate anxiety	Hesitation, fear expressions, partial cooperation	Enhanced communication, modeling, distraction	Improved cooperation, reduced distress
High anxiety	Crying, refusal, avoidance behavior	Gradual desensitization, environmental control, short sessions	Anxiety reduction over successive visits

## Data Availability

No new data were created or analyzed in this study. Data sharing is not applicable to this article.

## References

[1] Klingberg G., Broberg A.G. (2007). Dental fear/anxiety and dental behaviour management problems in children and adolescents: A review of prevalence and concomitant psychological factors. Int. J. Paediatr. Dent..

[2] Cianetti S., Lombardo G., Lupatelli E., Pagano S., Abraha I., Montedori A., Caruso S., Gatto R. (2017). Dental fear/anxiety among children and adolescents: A systematic review. Eur. J. Paediatr. Dent..

[3] Gao X., Hamzah S.H., Yiu C.K.Y., McGrath C., King N.M. (2013). Dental fear and anxiety in children and adolescents: Qualitative study using YouTube. J. Med. Internet Res..

[4] Klaassen M.A., Veerkamp J.S.J., Hoogstraten J. (2007). Dental fear, communication, and behavioural management problems in children referred for dental problems. Int. J. Paediatr. Dent..

[5] Porritt J., Buchanan H., Hall M., Gilchrist F., Marshman Z. (2013). Assessing children’s dental anxiety: A systematic review of current measures. Community Dent. Oral Epidemiol..

[6] American Academy of Pediatric Dentistry (2020). Behavior guidance for the pediatric dental patient. Pediatr. Dent..

[7] Merdad L., El-Housseiny A.A. (2017). Do children’s previous dental experience and fear affect their perceived oral health-related quality of life (OHRQoL)?. BMC Oral Health.

[8] Rajeswari S.R., Ramesh M.V. (2019). Effectiveness of cognitive behavioral play therapy and audiovisual distraction for management of preoperative anxiety in children. Int. J. Clin. Pediatr. Dent..

[9] Rodd H., Kirby J., Duffy E., Porritt J., Morgan A., Prasad S., Baker S., Marshman Z. (2018). Children’s experiences following a CBT intervention to reduce dental anxiety: One year on. Br. Dent. J..

[10] Shahnavaz S., Hedman-Lagerlöf E., Hasselblad T., Reuterskiöld L., Kaldo V., Dahllöf G. (2018). Internet-based cognitive behavioral therapy for children and adolescents with dental anxiety: Open trial. J. Med. Internet Res..

[11] Townend E., Dimigen G., Fung D. (2000). A clinical study of child dental anxiety. Behav. Res. Ther..

[12] Armfield J.M. (2010). How do we measure dental fear and what are we measuring anyway?. Oral Health Prev. Dent..

[13] Muhabes M., Al-Obaidi N. (2024). Understanding and addressing pediatric dental Anxiety: A contemporary overview. Al-Azhar J. Dent. Sci..

[14] Folayan M.O., Idehen E.E., Ojo O.O. (2004). The modulating effect of culture on the expression of dental anxiety in children: A literature review. Int. J. Paediatr. Dent..

[15] Armfield J.M., Heaton L.J. (2013). Management of fear and anxiety in the dental clinic: A review. Aust. Dent. J..

[16] Indriyani N.M.N., Albertina D., Montesinos D., Fedre R.W., Putri W. (2023). The impact of dental anxiety on oral health-related quality of life in children: A longitudinal study in Jakarta, Indonesia. Crown J. Dent. Health Res..

[17] Frankl S.N., Shiere F.R., Fogels H.R. (1962). Should the parent remain with the child in the dental operatory?. J. Dent. Child..

[18] Tiwari S., Kulkarni P., Agrawal N., Mali S., Kale S., Jaiswal N. (2021). Dental anxiety scales used in pediatric dentistry: A systematic review and meta-analysis. J. Contemp. Dent. Pract..

[19] Cuthbert M.I., Melamed B.G. (1982). A screening device: Children at risk for dental fears and management problems. J. Dent. Child..

[20] Peretz B., Bimstein E. (2000). The use of imagery suggestions during dental treatment in children. J. Dent. Child..

[21] Besiroglu-Turgut E., Kayaalti-Yuksek S., Bulut M. (2024). Evaluation of the relationship between dental anxiety and oral health status of mothers and their children. BMC Oral Health.

[22] Newton J.T., Asimakopoulou K., Daly B. (2012). The management of dental anxiety: Time for a sense of proportion?. Br. Dent. J..

[23] Kotsanos N., Arhakis A., Coolidge T. (2005). Parental presence versus absence in the dental operatory: A technique to manage the uncooperative child dental patient. Eur. J. Paediatr. Dent..

[24] Liu Y., Gu Z., Wang Y., Wu Q., Chen V., Xu X., Zhou X. (2019). Effect of audiovisual distraction on the management of dental anxiety in children: A systematic review. Int. J. Paediatr. Dent..

[25] Kain Z.N., Mayes L.C., O’Connor T.Z., Cicchetti D.V. (1996). Preoperative anxiety in children: Predictors and outcomes. Arch. Pediatr. Adolesc. Med..

[26] Hegazi F., Alghamdi N., Alhajri D., Alabdulqader L., Alhammad D., Alshamrani L., Bedi S., Sharma S. (2024). Association between Dental Fear and Children’s Oral Health-Related Quality of Life. Int. J. Environ. Res. Public Health.

[27] ten Berge M., Veerkamp J.S.J., Hoogstraten J., Prins P.J.M. (2002). Childhood dental fear in the Netherlands: Prevalence and normative data. Community Dent. Oral Epidemiol..

[28] Barry M., Alnami M., Alshobaili Y.T., Felemban O.M., Sabbagh H.J. (2025). Assessment of Dental Fear and Anxiety Tools for Children: A Review. Healthcare.

[29] Buchanan H., Niven N. (2002). Validation of a facial image scale to assess child dental anxiety. Int. J. Paediatr. Dent..

[30] Oliveira M.A., Vale M.P., Bendo C.B., Paiva S.M., Serra-Negra J.M. (2014). Dental Fear Survey: A cross-sectional study evaluating the psychometric properties of the Brazilian Portuguese version. Sci. World J..

[31] Klingberg G. (2008). Dental anxiety and behaviour management problems in paediatric dentistry—A review of background factors and diagnostics. Eur. Arch. Paediatr. Dent..

[32] Thribhuvanan L., Saravanakumar M.S., Anjana G. (2021). Influence of parental anxiety on children’s behaviour during their visits to dental clinic: A short clinical study. Bull. Natl. Res. Cent..

[33] Luoto A., Tolvanen M., Rantavuori K., Pohjola V., Lahti S. (2010). Can parents and children evaluate each other’s dental fear?. Eur. J. Oral Sci..

[34] Gustafsson A., Broberg A., Bodin L., Berggren U., Arnrup K. (2010). Dental behaviour management problems: The role of child personal characteristics. Int. J. Paediatr. Dent..

[35] Versloot J., Veerkamp J.S.J., Hoogstraten J. (2004). Children’s coping with pain during dental care. Community Dent. Oral Epidemiol..

[36] Blomqvist M., Ek U., Fernell E., Holmberg K., Westerlund J., Dahllöf G. (2013). Cognitive ability and dental fear and anxiety. Eur. J. Oral Sci..

[37] Shindova M.P., Belcheva A.B. (2021). Dental fear and anxiety in children: A review of the environmental factors. Folia Med..

[38] Cox I.C.J., Krikken J.B., Veerkamp J.S.J. (2011). Influence of parental presence on the child’s perception of, and behaviour, during dental treatment. Eur. Arch. Paediatr. Dent..

[39] Locker D. (2003). Psychosocial consequences of dental fear and anxiety. Community Dent. Oral Epidemiol..

[40] AlSarheed M. (2011). Children’s perception of their dentists. Eur. J. Dent..

[41] Suprabha B.S., Rao A., Choudhary S., Shenoy R. (2011). Child dental fear and behavior: The role of environmental factors in a hospital cohort. J. Indian Soc. Pedod. Prev. Dent..

[42] Farhat-McHayleh N., Harfouche A., Souaid P. (2009). Techniques for managing behaviour in pediatric dentistry: Comparative study of live modelling and tell-show-do based on children’s heart rates during treatment. J. Can. Dent. Assoc..

[43] Patil R.U., Onkari P.S., Gurunathan D. (2024). Effectiveness of audiovisual distraction in reducing children’s anxiety for pain during medical and dental treatments: A systematic review and meta-analysis. Saudi J. Med. Med. Sci..

[44] Wright G.Z., Kupietzky A. (2014). Behavior Management in Dentistry for Children.

[45] Roberts J.F., Curzon M.E.J., Koch G., Martens L.C. (2010). Behaviour management techniques in paediatric dentistry: A review. Eur. Arch. Paediatr. Dent..

[46] Yan X., Yan Y., Cao M., Xie W., O’Connor S., Lee J.J., Ho M.-H. (2023). Effectiveness of virtual reality distraction interventions to reduce dental anxiety in paediatric patients: A systematic review and meta-analysis. J. Dent..

[47] Khandelwal D., Tyagi R., Khatri A., Kalra N. (2025). Effectiveness of audiovisual distraction in the management of dental anxiety in 5–8 year old children: An observational study. Indian J. Behav. Sci..

